# *In Vitro* Antioxidant Activities of Three Red Wine Polyphenols and Their Mixtures: An Interaction Study

**DOI:** 10.3390/molecules171214336

**Published:** 2012-12-03

**Authors:** Elena Kurin, Pavel Mučaji, Milan Nagy

**Affiliations:** Department of Pharmacognosy and Botany, Faculty of Pharmacy, Comenius University, Odbojárov 10, Bratislava 83232, Slovakia; E-Mails: elena.kurin@gmail.com (E.K.); mucaji@fpharm.uniba.sk (P.M.)

**Keywords:** antioxidants, quercetin, resveratrol, caffeic acid, synergy, antagonism

## Abstract

The well-known antioxidant activity of red wine is explained mostly by its polyphenols content, where the final effect is based on the wine components’ interaction. The aim of our work was the study of the interaction of three red wine polyphenols—quercetin, resveratrol and caffeic acid—alone and in their equimolar binary and ternary mixtures in different antioxidant/scavenging assays (inhibition of 2-deoxy-D-ribose degradation by hydroxyl radical, FRAP, Fe(III) reducing power, DPPH, ABTS and NO scavenging, respectively). Interaction analysis, based on median effect equation, was performed for the determination of synergy and/or antagonism. The obtained results indicate that the mutual interactions of tested polyphenols in their mixtures are markedly different from each other, depending on the reaction mechanism of the assay used. The measured antioxidant activity of individual polyphenols is not a constant value when other substances are present in the mixture with this polyphenol. Interactions can cause the finally observed synergy/antagonism/additive effects without any possibility of predicting them from the known activities of single compounds. This “unpredictability” claim based on *in vitro* assay results should be very important in multiple systems and processes in Nature, where the interactions among compounds in mixtures need to be take into account.

## 1. Introduction

The importance of reactive oxygen species (ROS) and free radicals has attracted increasing attention over the past decades. ROS are continuously produced during normal physiologic events and in a healthy organism there should exist a balance between the production and inactivation of ROS by the functional antioxidant system. Under pathological conditions ROS are overproduced and if the endogenous antioxidant defenses are inadequate, the final result is oxidative stress. This leads to oxidative modifications of the cellular membranes or intracellular molecules [1]. In order to combat and neutralize the deleterious effects of ROS, various antioxidant strategies have evolved either by increasing the endogenous antioxidant enzyme defenses or by enhancing the non-enzymatic ones through dietary or pharmacological means. Dietary polyphenols have been reported to possess, among other effects, potent antioxidant activity by various endogenous and exogenous mechanisms [2].

Epidemiological studies and associated meta-analyses strongly suggest that long term consumption of foods rich in plant polyphenols offers some protection against the development of cancer, cardiovascular diseases, diabetes, osteoporosis and neurodegenerative diseases [3,4]. Red wine is rich in polyphenols (flavanols and their proanthocyanidin oligomers, anthocyanins, hydroxylated stilbenes such as resveratrol, flavonols such as kaempferol and quercetin, hydroxycinnamic acids such as *p*-hydroxy-coumaric and caffeic acid, as well as ellagitannins, ellagic acid). The total amount of polyphenols in red wines has been estimated to range from near 2,000 to about 6,000 mg/L [5]. The antioxidant effects of red wine and of its major polyphenols have been demonstrated in many experimental systems spanning the range from *in vitro* studies (human low-density lipoprotein, liposomes, macrophages, cultured cells) to investigations in healthy human subjects [6].

It is known that plants and polycomponent products of plants contain a wide diversity of specialized constituents, and this diversity presupposes a high likelihood of interactions [7]. Because the mode of action of complex mixtures from plants cannot be attributed to a single compound in most cases, additive or even synergistic interactions of combinations were postulated. Thus, synergy is present, if the effect seen by a combination of substances is greater than would have been expected from a consideration of individual contributions [8]. Synergy assessment has become a key area in phytomedicine research in recent years.

**Figure 1:**
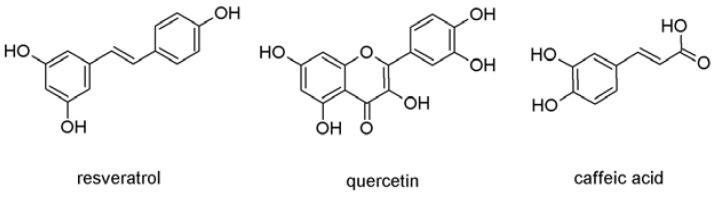
Structures of resveratrol, quercetin and caffeic acid.

Based on the abovementioned premises the aim of our work was the study of the interaction of three red wine polyphenols—quercetin (Q), resveratrol (R) and caffeic acid (C) (Figure 1)—alone and in their equimolar binary and ternary mixtures in different antioxidant/scavenging assays (inhibition of 2-deoxy-D-ribose degradation by hydroxyl radical, FRAP, reducing power, DPPH, ABTS and NO scavenging, respectively). Interaction analysis, based on the median effect equation, was performed for the determination of synergy and/or antagonism.

## 2. Results and Discussion

### 2.1. Antioxidant Effects of Single Wine Polyphenols and Their Mixtures

Wine is one of the most important sources of dietary polyphenolic antioxidants, including a large variety of both flavonoid and non-flavonoid compounds [9]. Two chemical processes shared by the majority of polyphenols are responsible for their antioxidant efficiency: hydrogen atom transfer (HAT) and electron transfer (ET) [10]. An indirect effect of polyphenols in a redox system lies in their ability to chelate transition metals (Fe, Cu) and thus directly reduce the rate of Fenton's reactions and prevent oxidation caused by highly reactive hydroxyl radicals [11].

We first tested the behavior of the three single wine polyphenols in seven different antioxidant assays (Table 1). Generally, and as expected, we confirmed that the polyphenols’ activities vary depending upon their structure and the mechanism of the assay. Resveratrol showed the lowest inhibitory activity in each assay used, except the ABTS one. Its effect was from 1.7- to 50-fold weaker if compared with quercetin, and from 1.9- to 21.8-fold less compared with caffeic acid. In three assays, SRD, NRD and FRAP, we observed that the ranking of the activity decrease is always in the order Q > C > R.

**Table 1:** Median inhibitory activities (IC_50_) and their correlation coefficients *r* of single polyphenols in different antioxidant assays.

	SRD		NRD		FRAP		RP		DPPH		ABTS		NO	
Sample	IC_50_ (µM)	*r*	IC_50_ (µM)	*r*	IC_50_ (µM)	*r*	IC_50_ (µM)	*r*	IC50 (µM)	*r*	IC_50_ (µM)	*r*	IC_50_ (µM)	*r*
Quercetin	3.82	0.95	3.61	0.97	55.08	0.99	253.51	0.98	4.36	0.99	2.87	0.97	54.94	0.94
Resveratrol	192.53	0.98	107.08	0.98	162.09	0.99	780.42	0.99	98.02	0.97	3.98	0.96	95.22	0.96
Caffeic acid	9.93	0.99	7.79	0.97	77.73	0.94	132.29	0.99	4.48	0.99	6.82	0.97	48.39	0.97

Median inhibitory concentrations (IC_50_) were calculated using the CompuSyn software for site-specific hydroxyl radical-mediated 2-deoxy-D-ribose degradation (SRD) and nonsite-specific hydroxyl radical-mediated 2-deoxy-D-ribose degradation (NRD) assays; ferric reducing/antioxidant assay (FRAP) assay, ferric ion reducing power (RP) assay, 2,2-diphenyl-1-picrylhydrazyl (DPPH) scavenging, 2,2'-azinobis-(3-ethylbenzothiazoline-6-sulfonic acid) (ABTS) scavenging and nitric oxide (NO) scavenging assays.

Comparing results with two Fenton’s reaction based assays, site-specific hydroxyl radical-mediated 2-deoxy-D-ribose degradation (SRD) characterizes hydroxyl radical scavenging, while nonsite-specific hydroxyl radical-mediated 2-deoxy-D-ribose degradation (NRD) additionally also involves a ferrous ion chelation mechanism [12]. One can only see small differences in the efficacy of quercetin and caffeic acid, respectively. This means their dominant reaction mechanism in both assays is the hydroxyl radical scavenging one, with only a small contribution of ferrous ion binding to the catecholic moiety of quercetin or caffeic acid. This explanation is in accord with a recent study, where it was suggested that the redox properties of quercetin in the H_2_O_2_/Fe(III)/ascorbic acid/2-deoxy-D-ribose system are more responsible for the observed antioxidative effect than the chelation of iron [13]. In contrast, the IC_50_ value of resveratrol in the SRD assay is about 1.8-fold higher than in the NRD one. This difference could be caused by increased resveratrol requirement/consumption in the former assay due to two concurrently running processes: poor hydroxyl radical scavenging and not very effective ferrous ion chelation [14,15]. Of note, in the NRD assay the Fe(III) chelation by the tested compounds is prevented by previous addition of EDTA, a chelating agent.

Our FRAP assay results are in an agreement with a previously published study [16]. This method shares a similar mechanism feature (an electron transfer) with the reducing power assay (RP)—Fe(III) reduction to Fe(II) by the tested compounds. Markedly different results can be explained by the differing test conditions: the ferric ion reduction in FRAP assay runs in a buffer at pH = 3.6, while no buffer (pH ~ 7) is used in RP one. Upon binding of quercetin or caffeic acid (bidentate ligands) to Fe(III), each polyphenol can reduce it to Fe(II). The polyphenol is oxidized to a semiquinone during this process. At low pH, the semiquinone ligand is protonated and is therefore a neutral ligand [17]. Then, a bidentate semiquinone can reduce another Fe(III) upon creating a quinone. Such a reaction occurs much more slowly around pH 7 [18]. Based on this mechanism, a monodentate ligand, e.g., resveratrol, cannot create semiquinones, and does not act as efficiently as bidentate ligands (e.g., quercetin, caffeic acid). Thus, very high IC_50_ values for resveratrol in the FRAP and RP assays are obvious (Table 1). The activity ranking switch for quercetin and caffeic acid, respectively, is probably related to the significant change of logD = 1.3 for caffeic acid at pH = 3.6 (FRAP assay) to logD = −1.5 at pH ~ 7 (RP assay). This change lead to a decrease of the existing neutral form of caffeic acid in the FRAP assay (~88%) to 0% in RP one, where the anionic form is present exclusively. Quercetin only exists in neutral form at the described pH conditions. Different electronic status may strongly influence the reactivity of both polyphenols. Nonetheless, this process of iron reduction is often attributed to both antioxidant and prooxidant activity of these compounds. Reduction of Fe(III) generates Fe(II) that can participate in the Fenton reaction and cause ROS generation [15]. The slower activity of polyphenols in these Fe(III) → Fe(II) assays could be even protective in some prooxidant cases.

The DPPH assay mechanism, when measured in a protic solvent, is also based on an electron transfer [19]. Thus, the tested compounds’ DPPH activity ranking is identical with their FRAP one, another ET-based method, and in correlation with the previous study [20].

The last ET-based method in our study was the ABTS cation radical scavenging assay. In this case, resveratrol was not the weakest antioxidant, as in all other assays used. Our established activity ranking Q > R > C is identical to that previously described [21].

NO scavenging is believed to run *via* a redox, but not a free radical mechanism [22]. A recent study has shown that (+)-catechin, (−)-epicatechin and especially quercetin have better ability to scavenge nitric oxid than resveratrol [23]. As the catechol group is a basic requirement for this reaction [24], the better efficacy of quercetin and caffeic acid compared to resveratrol is obvious. Slightly higher activity of caffeic acid than quercetin could be a result of similar logD values, as mentioned for the RP assay.

### 2.2. Interaction Analysis of Antioxidant Activities of Polyphenolic Mixtures

We prepared equimolar binary and ternary mixtures and then evaluated their median inhibitory activities (Table 2). For each assay and each combination we calculated the contribution of a single compound to the mixture, therefore all changes in the mass proportion effect on the median activity can be seen. For example, in the NRD assay we needed separately 3.61 µM (*r *= 0.97) of quercetin or 107.08 µM (*r *= 0.98) of resveratrol to reach the median inhibition of 2-deoxy-D-ribose degradation. In an equimolar Q+R mixture we needed only 4.2 µM (*r* = 0.89) of mixture, what means 2.1 µM from each sample component. A sophisticated approach for interactions evaluation can be done by combination index (CI) and dose-reduction index (DRI) calculations [25]. We calculated CI values for each equimolar mixture and in each assay, and evaluated them as follows: CI < 1 indicates synergy, CI = 1 indicates an additive effect, and CI > 1 indicates antagonism. The theoretical base for CI and DRI calculations is given in the Section 3.10.

**Table 2:** Median inhibitory activities (IC_50_), the absolute contributing concentrations of the single compounds (µM), correlation coefficients *r* and dose-reduction index (DRI) of equimolar mixtures of polyphenols in different antioxidant assays.

	Mixture	IC_50_ (µM)	Absolute contributions (µM)	*r*	DRI
SRD	Q+R	13.6	6.8 + 6.8	0.99	0.6:28.3
	Q+C	4.8	2.4 + 2.4	0.98	1.6:4.2
	R+C	56.3	28.15 + 28.15	0.92	6.8:0.4
	Q+R+C	17.0	5.66 + 5.66 + 5.66	0.95	0.7:34.0:1.8
NRD	Q+R	4.2	2.1 + 2.1	0.89	1.7:51.4
	Q+C	2.8	1.4 + 1.4	0.97	2.6:5.5
	R+C	8.0	4.0 + 4.0	0.96	26.7:1.9
	Q+R+C	3.5	1.16 + 1.16 + 1.16	0.99	3.1:92.4:6.7
FRAP	Q+R	185.7	92.85 + 92.85	0.99	0.6:1.7
	Q+C	60.8	30.4 + 30.4	0.93	1.8:2.6
	R+C	97.8	48.9 + 48.9	0.99	3.3:1.6
	Q+R+C	73.3	24.43 + 24.43 + 24.43	0.98	2.3:6.6:3.2
RP	Q+R	231.0	15.5 + 15.5	0.99	2.2:6.8
	Q+C	122.7	61.35 + 61.35	0.99	4.1:2.2
	R+C	193.8	96.9 + 96.9	0.99	8.1:1.4
	Q+R+C	128.1	42.7 + 42.7 + 42.7	0.99	5.9:18.3:3.1
DPPH	Q+R	11.4	5.7 + 5.7	0.99	0.8:17.3
	Q+C	4.3	2.15 + 2.15	0.97	2.0:2.1
	R+C	10.4	5.2 + 5.2	0.99	19.0:0.9
	Q+R+C	7.6	2.53 + 2.53 + 2.53	0.98	1.7:38.7:1.8
ABTS	Q+R	13.6	6.8 + 6.8	0.99	1.4:2.0
	Q+C	4.8	2.4 + 2.4	0.98	1.1:2.7
	R+C	56.3	28.15 + 28.15	0.92	1.2:2.1
	Q+R+C	17.0	8.5 + 8.5	0.95	1.6:2.2:3.7
NO	Q+R	78.2	39.1 + 39.1	0.93	1.4:2.4
	Q+C	34.9	17.45 + 17.45	0.95	3.1:2.8
	R+C	41.0	20.5 + 20.5	0.97	4.6:2.4
	Q+R+C	36.4	12.13 + 12.13 + 12.13	0.92	4.5:7.8:4.0

**Figure 2:**
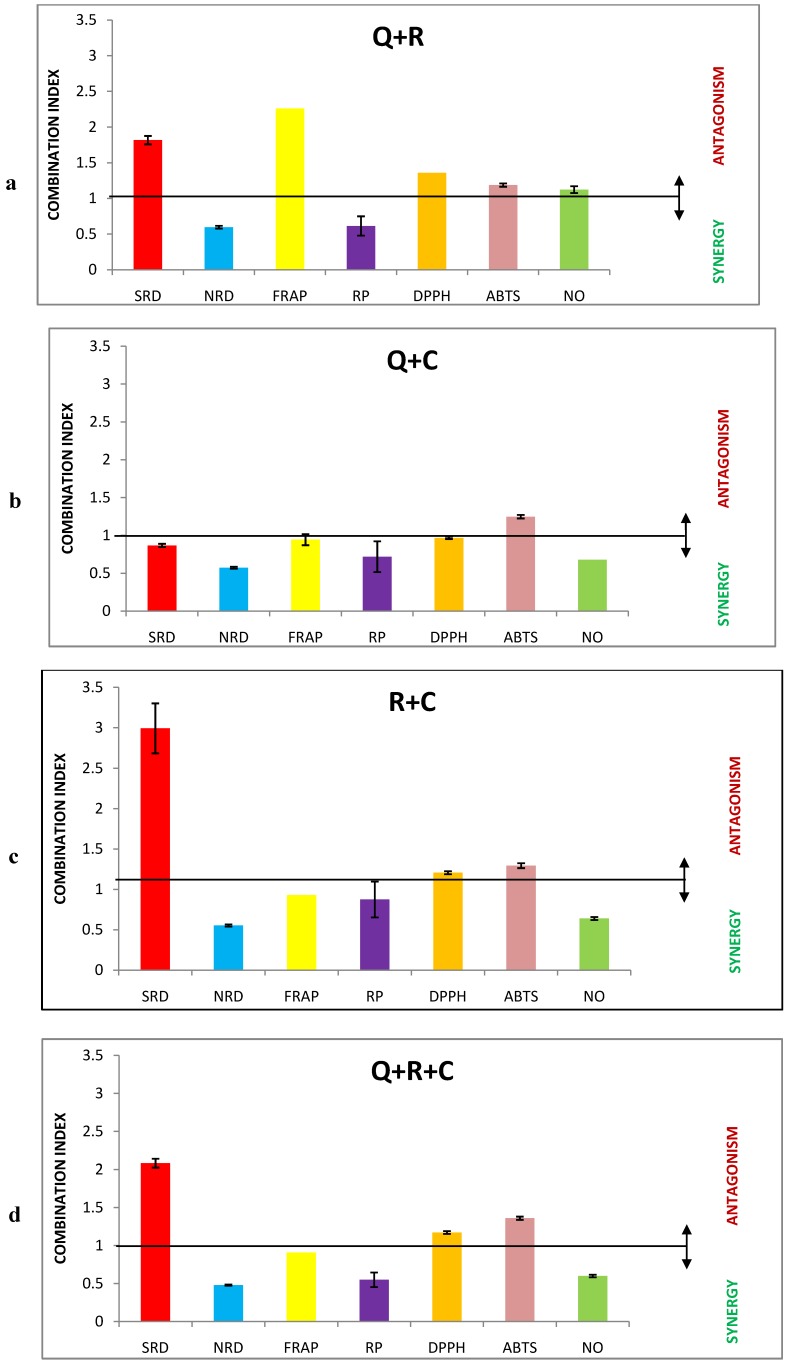
(**a**) Interaction analysis of quercetin (Q) and resveratrol (R) equimolar mixtures in different antioxidant assays; (**b**) Interaction analysis of quercetin (Q) and caffeic acid (C) equimolar mixtures in different antioxidant assays; (**c**) Interaction analysis of resveratrol (R) and caffeic acid (C) equimolar mixtures in different antioxidant assays; (**d**) Interaction analysis of quercetin (Q), resveratrol (R) and caffeic acid (C) equimolar mixtures in different antioxidant assays. Each value error bars are expressed as ± SDA (an iterative sequential deletion of one dose of a drug at a time for repetitive CI calculations, as calculated by CompuSyn software). CI value indicates synergy, additivity or antagonism, when it is <, = or > than 1. Antioxidant assays are abbreviated as site-specific hydroxyl radical-mediated 2-deoxy-D-ribose degradation (SRD) and nonsite-specific hydroxyl radical-mediated 2-deoxy-D-ribose degradation (NRD) assays; ferric ion reducing/antioxidant power (FRAP) and ferric ion reducing power (RP) assay, 2,2-diphenyl-1-picrylhydrazyl scavenging (DPPH), 2,2'-azinobis-(3-ethylbenzothiazoline-6-sulfonic acid) (ABTS) scavenging and nitric oxide (NO) scavenging assays.

Median inhibitory concentrations (IC_50_), the absolute contributing concentrations of the single compounds, correlation coefficients *r* and dose-reduction index (DRI) of equimolar mixtures of polyphenols (Q—quercetin, R—resveratrol, C—caffeic acid). Results were calculated using the CompuSyn software in site-specific hydroxyl radical-mediated 2-deoxy-D-ribose degradation (SRD) and nonsite-specific hydroxyl radical-mediated 2-deoxy-D-ribose degradation (NRD) assays; ferric ion reducing/antioxidant power (FRAP) assay, ferric ion reducing power (RP) assay; 2,2-diphenyl-1-picrylhydrazyl (DPPH) scavenging, 2,2'-azinobis-(3-ethylbenzothiazoline-6-sulfonic acid) (ABTS) scavenging and nitric oxide (NO) scavenging assays. It is illustrative (Figure 2) that mixing of the tested polyphenols led to the different types of interaction.

Mixture Q+R showed antagonistic behavior in four assays (SRD, FRAP, DPPH, ABTS), in one test (NO) an additive effect was determined, and in the remaining two (NRD, RP) synergy was observed. Mixture Q+C acted synergically in six assays, except the ABTS one, which was antagonistic.

Previous study has shown that mixtures of quercetin and caffeic acid act slightly antagonistically in the linoleic acid system in an aqueous dispersion with 2,2'-azobis (2-amidinopropane) dihydrochloride (AAPH). The authors explained this antagonistic effect between two antioxidants as an effect whereby the more efficient molecule regenerates the less efficient one; in this case caffeic acid is regenerated by quercetin [26]. Mixture R+C acted antagonistically in three tests (SRD, DPPH, ABTS), and in the remaining four (NRD, NO, FRAP, RP) we observed synergy. Finally, the behavior of the mixture Q+R+C was similar to the R+C one. It is clear that there is no relationship between interaction type and similar reaction mechanism of the assay, e.g., ET-based reactions (SRD and NRD assays) or ferric ion reduction-based ones (FRAP and RP) showed opposite interaction quality (antagonism *vs*. synergy) based on different mixture compositions. Resveratrol occurrence in the tested mixtures seems to be a significant negative factor for the SRD, DPPH and ABTS assays, and a positive one for NRD and RP tests, respectively. Analogically, caffeic acid presence can negatively affect the final mixture behavior in the ABTS test. We cannot offer an exact explanation for these effects at this time.

Mean CI values were used for previous comparisons. Using SDA deviation values (as shown in Figure 2) could lead in some cases to the following results: for Q+R mixture, five antagonisms occurred: SRD, FRAP, DPPH, ABTS, NO; no additive behavior; two synergies (NRD, RP). Mixture Q+C: no differences with the abovementioned results. Mixture R+C: three antagonisms (SRD, DPPH, ABTS), one additive behavior (RP), three synergies (NRD, NO, FRAP). Finally, mixture Q+R+C showed no behavior changes. Again, there are none of the abovementioned relationships. Generally, one cannot predict the type interaction based on some theoretical considerations without any measurements.

For better understanding of single substances’ participation in a mixture, we also evaluated DRI values (Table 2). The DRI is a measure of how many-fold the dose of each drug in a synergistic combination may be reduced at a given effect level (the median effect in our study) compared with the doses of each drug alone. For example, in the NRD assay we needed a 1.7-fold lower dose of quercetin and a 51.4-fold lower dose of resveratrol in an equimolar mixture to achieve the same median activity as measured for the single substances. In other words, IC_50_ values were decreased from 3.61 µM for quercetin alone to 2.1 µM for quercetin in an equimolar Q+R mixture and from 107.08 µM for resveratrol alone to 2.1 µM for resveratrol in this equimolar Q+R mixture. The highest decrease of a dose needed for the same median effect, when single compounds and their mixtures were compared, was calculated for resveratrol and its mixtures in assays where extremely low resveratrol activity was observed (NRD, SRD and DPPH). Different mutual interactions of polyphenols in binary and ternary mixture(s), when we evaluated one concrete assay, always caused different DRI values for each polyphenol in its one ternary mixture and two binary ones. For example, DRIs for resveratrol in NRD assay are: 51.4 in mixture Q+R, 26.7 in mixture R+C, and 92.4 in mixture Q+R+C. Then, calculated IC_50_ values (µM) for resveratrol are: 107.08 µM:51.4 = 2.1µM (mixture Q+R), 107.08:26.7 = 4.0 (mixture R+C), and 107.08:92.4 = 1.2 (mixture Q+R+C). Analogously, in the same assay, DRIs for quercetin (Q+R, Q+C, and Q+R+C mixtures evaluated) are 1.7, 2.6, and 3.1, respectively, and their corresponding IC_50_s (µM) are 2.1, 1.4, and 1.2, respectively. Note, all the mentioned values are presented after rounding to one decimal place.

This variability of all DRIs in the concrete assays means there are no uniform interactions between particular components for all analyzed mixture compositions. Combining one polyphenol compound (e.g., resveratrol) separately with another one (either quercetin or caffeic acid) will lead to quite different mutual “binary” interactions (and different “binary” DRIs) in the respective binary mixture. Adding one compound (e.g., resveratrol) to the binary mixture of another two polyphenols (quercetin and caffeic acid) will cause a significant change in the original mutual “binary” interaction, which will lead to changes to the original “binary” DRIs and the creation of “ternary” DRIs. These subsequently will cause various IC_50_ values for all evaluated compounds, when compared to their original IC_50_s.

## 3. Experimental

### 3.1. Instrument and Software

All spectrophotometric measurements were performed on an Infinite M200 microplate reader (Tecan AG, Grödig/Salzburg, Austria) using 96-well microplates (UV-Star F-bottom chimney well μCLEAR, Greiner Bio-One, Frickenhausen, Germany). Interaction analysis and IC_50_ values were determined using CompuSyn software version 1.0.1., ComboSyn Inc., Paramus, NJ, USA. LogD values and % of anionic/neutral forms of resveratrol, quercetin and caffeic acid were calculated using ACD/ADME Suite 5.0.7 (Build 1339, 2010; Advanced Chemistry Development, Inc., Toronto, ON, Canada).

### 3.2. Chemicals

Resveratrol (R; ≥99% purity), quercetin (Q; ≥98% purity), caffeic acid (C; ≥98% purity) and other used chemicals (always the highest purity grade available) were purchased from Sigma-Aldrich (Steinheim, Germany).

### 3.3. Site-Specific Hydroxyl Radical-Mediated 2-Deoxy-D-ribose Degradation (SRD)

Inhibition of site-specific hydroxyl radical-mediated 2-deoxy-D-ribose degradation was measured according to Halliwell with slight adaptations [12]. The reaction mixture contained 6 μL of sample dissolved in dimethylsulfoxide (DMSO) and made up to 100 μL with distilled water. 2-Deoxy-D-ribose (500 μL, 5.6 mM, in KH_2_PO_4_-NaOH buffer, pH 7.4), FeCl_3_ (200 μL, 100 μM), H_2_O_2_ (100 μL, 1.0 mM) and ascorbic acid (100 μL, 1.0 mM) were added to the reaction mixture and after 30 min incubation at 50 °C CCl_3_COOH (1 mL, 2.8%) and thiobarbituric acid (1 mL, 1.0%) were added, vortexed and heated at 95 °C for 30 min. The extent of oxidation was estimated from the solution absorbance at 532 nm. From dose-response relationship IC_50_ was calculated [we took the contribution of DMSO (0.2% in final samples) to the reaction into consideration in our calculations].

### 3.4. Nonsite-Specific Hydroxyl Radical-Mediated 2-Deoxy-D-Ribose Degradation (NRD)

Inhibition of nonsite-specific hydroxyl radical-mediated degradation was carried out according to Halliwell with slight adaptations [12]. The reaction mixture contained 6 μL of sample dissolved in DMSO and made up to 100 μL with distilled water. 2-Deoxy-D-ribose (500 μL, 5.6 mM, in KH_2_PO_4_-NaOH buffer, pH 7.4), premixed (200 μL, 1:1, v/v) FeCl_3_ (100 μM) and EDTA (104 μM), H_2_O_2_ (100 μL, 1.0 mM) and ascorbic acid (100 μL, 1.0 mM) were mixed in and after 30 min incubation at 50 °C CCl_3_COOH (1 mL, 2.8%) and thiobarbituric acid (1 mL, 1.0%) were added, vortexed and heated at 95 °C for 30 min. The extent of oxidation was estimated from the solution absorbance at 532 nm. From the dose-response relationship the IC_50_ was calculated [we took the contribution of DMSO (0.2% in final samples) to reaction into consideration in our calculations].

### 3.5. Ferric Ion Reducing Antioxidant Power Assay (FRAP)

The FRAP assay with slight modifications according to Benzie and Strain was used [27]. FRAP solution was prepared from acetate buffer (10 mL, 300 mM, pH 3.6) mixed with FeCl_3_ × 6H_2_O (1 mL, 20 mM) and 2,4,6-tris-(2-pyridyl)-*s*-triazine (1 mL, 10 mM dissolved in 40 mM HCl). Ten μL of sample diluted in ethanol was mixed with FRAP solution (240 μL). Changes in absorbance were measured after 5 min at 593 nm and from the dose-response relationship the IC_50_ value was calculated.

### 3.6. Ferric Ion Reducing Power Assay (RP)

The method according to Tian and Hua [28] with slight modifications was used. Sample (0.5 mL) diluted in water was mixed with K_3_Fe(CN)_6_(0.5 mL, 1%). After 20 min of incubation at 50 °C CCl_3_COOH (0.5 mL, 10%) was added. The mixture was centrifuged at 3,000 × *g* for 10 min. The upper layer of the solution (1.0 mL) was mixed with distilled water (1 mL) and FeCl_3_ (0.2 mL, 0.1%). Changes in absorbance were measured at 700 nm and from the dose-response relationship an IC_50_ was calculated.

### 3.7. DPPH Scavenging Assay

The DPPH radical scavenging assay was determined according to Blois [29] with slight adaptations. Briefly, stable 2,2-diphenyl-1-picrylhydrazyl (DPPH) radical was *ex tempore* dissolved in methanol. DPPH (225 µL, 55 µM) was mixed with sample dissolved in methanol (25 µL, 12.5–100 µM). Changes in absorbance were measured after 30 min at 517 nm and from the dose-response relationship the IC_50_ value was calculated.

### 3.8. ABTS Scavenging Assay

The ABST radical scavenging assay was determined according to Re [30] with slight adaptations. Briefly, 7 mM 2,2'-azinobis-(3-ethylbenzothiazoline-6-sulfonic acid) (ABTS) aqueous solution was mixed in equimolar ratio with 2.45 mM K_2_S_2_O_8_, allowing the mixture to stand in the dark at room temperature for 24 hours before use. After that the ABTS solution was diluted with ethanol (1.1 mL) to a final volume of 50 mL. Sample diluted in ethanol (2.5 µL, 62.5–200 µM) was added to ABTS solution (247.5 µL). Changes in absorbance were measured after 6 min at 734 nm and from the dose-response relationship the corresponding IC_50_ was calculated.

### 3.9. NO Scavenging Assay

The NO scavenging ability of samples was measured according to Marcocci [31]. Sodium nitroprusside (SNP, 0.1 mL, 100 mM in PBS, pH 7.4) was mixed with sample dissolved in water (1.9 mL, 1.6–210.5 µM). After 150 min incubation at 25 °C an equivolume mixture of sample with SNP and Griess reagent (1% sulfanilamide in 5% H_3_PO_4_ and 0.1% naphthylethylenediamine dihydrochloride) was prepared. Changes in absorbance were measured after 10 min at 546 nm and from the dose-response relationship the IC_50_ was calculated.

### 3.10. Statistical and Interaction Analysis

All measurements were performed in four replicates. The quantification of interaction as a synergism or antagonism was done by the general equation (1) for *n*-drug combination at x % inhibition according to Chou [25] using combination index (CI) for interaction interpretation:

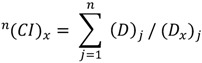
(1)
where^ n^(CI)_x_ is the sum of the dose of *n* drugs that exerts x % inhibition in a combination. In the denominator (D_x_) is for D “alone” that inhibits a system x %. If CI value is equal, smaller or greater to 1, an additive, synergistic or antagonistic effect is indicated. Sequential deletion analysis (SDA), an iterative sequential deletion of one dose of a drug at a time for repetitive CI calculations and *r *value analysis, which represents the conformity parameter for goodness of fit to the median-effect principle of the mass-action law [25], were calculated using a median-effect analysis by CompuSyn software.

The dose-reduction index (DRI) means how many-fold the dose of each drug in a synergic combination could be reduced at a given effect level compared with the doses of each drug alone. The DRI value for each corresponding drug was given for n-drug combinations, Equation (2):

(DRI)_1_ = (D_x_)_1_/(D_1_); (DRI)_2_ = (D_x_)_2_/(D_2_); … ; *etc*. (2)

Value of DRI > 1 indicates a favorable dose reduction, and the higher DRI value indicates the higher dose reduction for a given therapeutic effect, but does not necessarily always indicate synergism [25].

## 4. Conclusions

Summarizing all above discussed results one can say that the antioxidant “quality” of tested polyphenols differs markedly when tested separately and in their mixtures. This means that the antioxidant activity of each polyphenol is not a constant value as measured individually when other substances are present (and interact) with this polyphenol in the same antioxidant assay. Interactions can cause all types of final measured effects (synergy/antagonism/additive effect) without any possibility of predicting them from the known activities of single compounds. This “unpredictability” claim based on our *in vitro* assays results should be the motivation for a closer study of *in vivo* systems and processes, where the interactions among compounds always take place in a mixture.

## References

[1] Gülçin I. (2006). Antioxidant activity of caffeic acid (3,4-dihydroxycinnamic acid). Toxicology.

[2] Obrenovich M.E., Nair N.G., Beyaz A., Aliev G., Reddy V.P. (2010). The role of polyphenolic antioxidants in health, disease, and aging. Rejuvenation Res..

[3] Pandey K.B., Rizvi S.I. (2009). Plant polyphenols as dietary antioxidants in human health and disease. Oxid. Med. Cell. Longev..

[4] Hanhineva K., Törrönen R., Bondia-Pons I., Pekkinen J., Kolehmainen M., Mykkänen H., Poutanen K. (2010). Impact of dietary polyphenols on carbohydrate metabolism. Int. J. Mol. Sci..

[5] Quideau S., Deffieux D., Douat-Casassus C., Pouységu L. (2011). Plant polyphenols: Chemical properties, biological activities, and synthesis. Angew. Chem. Int. Ed. Engl..

[6] Soleas G.J., Diamandis E.P., Goldberg D.M. (1997). Wine as a biological fluid: History, production, and role in disease prevention. J. Clin. Lab. Anal..

[7] Biavatti M.W. (2009). Synergy: An old wisdom, a new paradigm for pharmacotherapy. Braz. J. Pharm. Sci..

[8] Herrmann F., Wink M. (2011). Synergistic interactions of saponins and monoterpenes in HeLa cells, Cos7 cells and in erythrocytes. Phytomedicine.

[9] Alén-Ruiz F., García-Falcón M.S., Pérez-Lamela M.C., Martínez-Carballo E., Simal-Gándara J. (2009). Influence of major polyphenols on antioxidant activity in Mencía and Brancellao red wines. Food Chem..

[10] Huang D., Ou B., Prior R.L. (2005). The chemistry behind antioxidant capacity assays. J. Agric. Food Chem..

[11] Tsao R. (2010). Chemistry and biochemistry of dietary polyphenols. Nutrients.

[12] Halliwell B., Gutteridge J.M.C., Aruoma O. (1987). The deoxyribose method: A simple “test tube” assay for determination of rate constants for reactionsof hydroxyl radicals. Anal. Biochem..

[13] Chobot V. (2010). Simultaneous detection of *pro*- and antioxidative effects in the variants of the deoxyribose degradation assay. J. Agric. Food Chem..

[14] Belguendouz L., Fremont L., Linard A. (1997). Resveratrol inhibits metal ion-dependent and independent peroxidation of porcine low-density lipoproteins. Biochem. Pharmacol..

[15] Perron N.R., Brumaghim J.L. (2009). A review of the antioxidant mechanisms of polyphenol compounds related to iron binding. Cell Biochem. Biophys..

[16] Pulido R., Bravo L., Saura-Calixto F. (2000). Antioxidant activity of dietary polyphenols as determined by a modified ferric reducing/antioxidant power assay. J. Agric. Food Chem..

[17] Hider R.C., Liu Z.D., Khodr H.H. (2001). Metal chelation of polyphenols. Methods Enzymol..

[18] Jameson G.N.L., Linert W. (2001). The oxidation of 6-hydroxydopamine in aqueous solution. Part 3. Kinetics and mechanism of the oxidation with iron(III). J. Chem. Soc.Perkin Trans. 2.

[19] Litwinienko G., Ingold K.U. (2003). Abnormal solvent effects on hydrogen atom abstractions. 1. The reactions of phenols with 2,2-diphenyl-1-picrylhydrazyl (dpph*) in alcohols. J. Org. Chem..

[20] Villano D., Fernández-Pachón M.S., Moyá M.L., Troncoso A.M., García-Parrilla M.C. (2007). Radical scavenging ability of polyphenolic compounds towards DPPH free radical. Talanta.

[21] Rice-Evans C.A., Miller N.J., Paganga G. (1996). Structure-antioxidant activity relationship of flavonoids and phenolic acids. Free Radic. Biol. Med..

[22] Sueishi Y., Hori M., Kita M., Kotake Y. (2011). Nitric oxide (NO) scavenging capacity of natural antioxidants. Food Chem..

[23] Číž M., Pavelková M., Gallová L., Králová J., Kubala L., Lojek A. (2008). The influence of wine polyphenols on reactive oxygen and nitrogen species production by murine macrophages RAW 264.7. Physiol. Res..

[24] De la Puerta R., Martínez Domínguez M.E., Ruíz-Gutíerrez V., Flavill J.A., Hoult J.R. (2001). Effects of virgin olive oil phenolics on scavenging of reactive nitrogen species and upon nitrergic neurotransmission. Life Sci..

[25] Chou T.C. (2006). Theoretical basis, experimental design, and computerized simulation of synergism and antagonism in drug combination studies. Pharmacol. Rev..

[26] Peyrat-Maillard M.N., Cuvelier M.E., Berset C. (2003). Antioxidant activity of phenolic compounds in 2,2'-azobis (2-amidinopropane) dihydrochloride (AAPH)-induced oxidation: Synergistic and antagonistic effects. J. Am. Oil Chem. Soc..

[27] Benzie I.F., Strain J.F. (1996). The ferric reducing ability of plasma (FRAP) as a measure of “antioxidant power”: The FRAP assay. Anal. Biochem..

[28] Tian B., Hua Y. (2005). Concentration dependence of prooxidant and antioxidant effects of aloin and aloe-emodin on DNA. Food Chem..

[29] Blois M.S. (1958). Antioxidant determination by the use of a stable free radical. Nature.

[30] Re R., Pellegrini N., Proteggente A., Pannala A., Yang M., Rice-Evans C. (1999). Antioxidant activity applying an improved ABTS radical cation decolorization assay. Free Radic. Biol..

[31] Marcocci L., Maguire J.J., Droy-Lefaix M.T., Packer L. (1994). The nitric oxide-scavenging properties of *Ginkgo biloba* extract EGB 761. Biochem. Biophys. Res. Commun..

